
JOURNAL INFORMATION
==============================
NLM Title Abbreviation: Medizinrecht
Journal ID: Medizinrecht
NLM Journal ID: 8309354

Medizinrecht

ISSN: 0723-8886
EISSN: 1433-8629

ARTICLE INFORMATION
==============================
PMCID: PMC9778455
PMID: 36575717
DOI: 10.1007/s00350-022-6356-x
Article ID: 6356
Article version: 1
Subjects: Aufsätze

Zur Rechtskonformität des Erfordernisses eines Lichtbildes für die eGK unter besonderer Berücksichtigung des Rechts auf informationelle Selbstbestimmung sowie der Vorgaben der DSGVO (Teil II)

Lorenz Luisa
grid.5155.4 0000 0001 1089 1036 FB 07, Universität Kassel, Henschelstraße 4, 34127 Kassel, Deutschland
Electronic publication date: 2022 Dec 22
Print publication date: 2022
Volume: 40
Issue: 12
First page: 1000
Last page: 1005
Copyright: © Der/die Autor(en) 2022
License: Open Access Dieser Artikel wird unter der Creative Commons Namensnennung 4.0 International Lizenz veröffentlicht, welche die Nutzung, Vervielfältigung, Bearbeitung, Verbreitung und Wiedergabe in jeglichem Medium und Format erlaubt, sofern Sie den/die ursprünglichen Autor(en) und die Quelle ordnungsgemäß nennen, einen Link zur Creative Commons Lizenz beifügen und angeben, ob Änderungen vorgenommen wurden.
Die in diesem Artikel enthaltenen Bilder und sonstiges Drittmaterial unterliegen ebenfalls der genannten Creative Commons Lizenz, sofern sich aus der Abbildungslegende nichts anderes ergibt. Sofern das betreffende Material nicht unter der genannten Creative Commons Lizenz steht und die betreffende Handlung nicht nach gesetzlichen Vorschriften erlaubt ist, ist für die oben aufgeführten Weiterverwendungen des Materials die Einwilligung des jeweiligen Rechteinhabers einzuholen.
Weitere Details zur Lizenz entnehmen Sie bitte der Lizenzinformation auf http://creativecommons.org/licenses/by/4.0/deed.de.
License URL: https://creativecommons.org/licenses/by/4.0/

issue-copyright-statement: © C.H. Beck Verlag oHG, München und Springer-Verlag GmbH Deutschland, ein Teil von Springer Nature 2022

==============================

**Luisa Lorenz**

**Dieser Beitrag ist die Fortsetzung von Teil I in Heft 11.**

## IV. Detailfragen zum Lichtbilderfordernis der eGK

Obschon Versicherte einen gesetzlichen Anspruch auf die Ausstellung einer eGK haben, trifft sie – die Rechtmäßigkeit des Lichtbilderfordernisses vorausgesetzt – eine Obliegenheit an der Ausstellung der eGK durch Einreichung eines geeigneten Lichtbildes mitzuwirken[57], wobei jedenfalls allein die Pflicht zur Vorlage des Bildes nach gefestigter Rechtsprechung keine Grundrechtsverletzung darstellt[58].

Eine ausdrückliche Mitwirkungspflicht, ein Foto bestimmter Größe und Qualität für die eGK bereitzustellen, enthalten die §§ 60 ff. SGB I hingegen nicht[59]. Eine solche lässt sich jedoch aus dem Versicherungsverhältnis sowie den Normen über die eGK ableiten (v. a. § 291a Abs. 5 S. 1 SGB V). Das BSG spricht – mit Blick auf § 15 Abs. 2 SGB V – ferner von einer „Nachweisobliegenheit“[60]. Weil ohne Bildeinreichung keine eGK ausgestellt wird, wodurch keine Leistungen in Anspruch zu nehmen sind, geht der Gesetzgeber von einem faktischen Nutzungszwang der eGK mit Lichtbild aus[61].

Nach § 291a Abs. 5 SGB V ist die eGK mit einem Lichtbild des Versicherten zu versehen. Versicherte, die jünger als 15 Jahre sind (vgl. § 36 Abs. 1 S. 1 SGB I) sowie Versicherte, deren Mitwirkung bei der Erstellung des Lichtbildes nicht möglich ist, erhalten (verpflichtend) eine eGK ohne Lichtbild[62]. Letzteres betrifft etwa Personen, die nicht mobil sind, sich in Pflege befinden oder krank sind und daher nicht fotografiert werden können[63]. Diese Ausnahmen sind abschließend[64]. Die GKV ist daher nicht befugt, Versicherten in anderen Fällen eine eGK ohne Lichtbild auszustellen.

### *1. Anforderung(en) an das Lichtbild*

Noch immer enthält das Gesetz keine konkreten Bestimmungen zur Beschaffenheit des Lichtbildes (anders als beispielsweise im Passwesen, s. dort die untergesetzliche Regelung des § 7 PAuswV). Gemäß § 291b Abs. 6 SGB V wird das Nähere zur bundesweiten Verwendung der eGK als Versicherungsnachweis durch die Vertragspartner der Verträge nach § 87 Abs. 1 SGB V vereinbart. Damit verweist § 291b Abs. 6 SGB V auf den zwischen der Kassenärztlichen Bundesvereinigung und dem Spitzenverband Bund der Krankenkassen geschlossenen Bundesmantelvertrag-Ärzte (BMV-Ä)[65]. Dieser verweist in § 3 Abs. 1 seiner Anlage 4a seinerseits auf die geltenden Vorgaben der gematik, die in ihren Spezifikationen für die Beschaffenheit der Lichtbilder[66] die jeweils gültige Passmusterverordnung heran zieht. Letztere sieht in Anlage 3 u. a. vor, dass die Gesichtshöhe 70–80 Prozent des Lichtbildes einnehmen muss und allein die zu fotografierende Person ohne weitere Gegenstände abbilden darf.

### *2. Vereinbarkeit mit Art. 4 GG*

Versicherte können sich auch nicht unter Hinweis auf die Freiheit des religiösen Bekenntnisses, konkret ihrer individuellen Glaubensüberzeugung der Obliegenheit entziehen, Leistungen nur unter Vorlage einer eGK mit Lichtbild beanspruchen zu können. Die Regelung des § 291a Abs. 5 SGB V ist aus höchstrichterlicher Sicht mit höherrangigem Recht, namentlich mit Art. 4 GG, vereinbar[67].

Insoweit führte das BSG aus, dass die Sicherung der finanziellen Stabilität der GKV eine verfassungsimmanente Schranke der Religionsfreiheit sei[68]. Deren Finanzierbarkeit wäre in einem Sozialstaat ein überragend wichtiges Gemeinschaftsgut; ferner ein Gemeinwohlbelang von derart hoher Bedeutung, dass Maßnahmen, die ihr zu dienen bestimmt sind, auch dann gerechtfertigt sein könnten, wenn sie für Betroffene zu fühlbaren Einschränkungen führen.

Luisa Lorenz, Wissenschaftliche Mitarbeiterin,
Fachgebiet Öffentliches Recht, IT-Recht und Umweltrecht
von Prof. Dr. iur. Gerrit Hornung, LL.M.,
Universität Kassel, FB 07,
Henschelstraße 4, 34127 Kassel, Deutschland

57) Die Kosten der Erstellung des Lichtbildes sind vom Versicherten zu tragen, LSG Rheinland-Pfalz, Beschl. v. 20. 3. 2014 – L 5 KR 32/14 NZB –, juris.
58) Vgl. nur LSG Bad.-Württ., Beschl. v. 11. 7. 2018 – L 4 KR 4901/17 ER –, juris, Rdnr. 5 mit Verweis auf LSG Bad.-Württ., Beschl. v. 29. 8. 2016 – L 5 KR 2729/16 ER und BSG, Urt. v. 18. 11. 2014 – B 1 KR 35/13 R –, jew. juris; BVerfG, Beschl. v. 8. 6. 2016 – 1 BvR 864/15 –, n. v.
59) *Scholz*, in: BeckOK Sozialrecht, 66. Ed., Stand: 1. 9. 2022, § 291a SGB V, Rdnr. 6.
60) BSG, Urt. v. 18. 11. 2014 – B 1 KR 35/13 R –, juris, Rdnr. 14.
61) *Scholz*, in: BeckOK Sozialrecht, 66. Ed., Stand: 1. 9. 2022, § 291a SGB V, Rdnr. 6.
62) Ebenso *gematik*, Die Spezifikation der elektronischen Gesundheitskarte – Äußere Gestaltung, Stand: 2. 10. 2019, Version 3.10.0, S. 19: „Das Aufbringen eines Lichtbildes auf die eGK MUSS bei Versicherten, deren Mitwirkung bei der Erstellung des Lichtbildes nicht möglich ist, entfallen.“, „Das Aufbringen eines Lichtbildes auf die eGK KANN bei Kindern bis zur Vollendung des 15. Lebensjahres entfallen.“; kritisch zu dieser Altersregelung zu Recht *Hornung*, in: *Hänlein/Schuler*, SGB V, 6. Aufl. 2022, § 291a, Rdnr. 16.
63) BT-Dr. 15/4228, S. 28.
64) BSG, Urt. v. 18. 11. 2014 – B 1 KR 35/13 R –, juris, Rdnr. 19.
65) SG Hamburg, Beschl. v. 14. 7. 2020 – S 30 KR 1024/20 ER –, juris, Rdnr. 16 (noch zu § 291 Abs. 3 SGB V a. F.).
66) *Gematik*, Die Spezifikation der elektronischen Gesundheitskarte – Äußere Gestaltung, Stand: 2. 10. 2019, Version 3.10.0, S. 19.
67) BSG, Beschl. v. 19. 3. 2020 – B 1 KR 89/18 B –, juris, Rdnrn. 9, 19. Die Verfassungsbeschwerde gegen den Beschluss wurde nicht zur Entscheidung angenommen (BVerfG, 16. 4. 2021 – 1 BvR 1529/20). Eine vergleichbare Diskussion wurde bzw. wird bei Personalausweisdokumenten geführt. § 7 Abs. 3 S. 4 PAuswV kann dabei als Versuch des Gesetzgebers verstanden werden, das Spannungsfeld grundrechtskonform aufzulösen. Im Übrigen stellt sich auch hier die Frage, wann der Schutzbereich des Art. 4 GG eröffnet ist. Dazu, ob die Kirche des Fliegenden Spaghettimonsters Deutschland e. V. eine Religions- oder Weltanschauungsgemeinschaft ist, VG Potsdam, Urt. v. 13. 11. 2016 – VG 8 K 4253/13 –, juris.
68) BSG, Beschl. v. 19. 3. 2020 – B 1 KR 89/18 B –, juris, Rdnr. 11 mit Verweis auf die einschlägige Rechtsprechung des BVerfG.

Eventuell entgegenstehende religiöse Gründe müssen durch geeignete Tatsachen hinreichend glaubhaft gemacht werden; die Verweigerung mit einem pauschalen Hinweis auf „religiöse Gründe" reicht nicht aus[69]. Eine gänzliche Befreiung vom Lichtbilderfordernis aus religiösen Gründen wird abgelehnt[70].

Ausdrücklich offen ließ das BSG demgegenüber, ob Versicherte aus religiösen Gründen Abweichungen von der Gestaltung des Lichtbildes beanspruchen könnten[71]. Nachdem die Klägerin die Ausstellung gänzlich ohne Lichtbild begehrte, stelle sich die Frage, ob und in welchem Umfang sie darlegen müsse, dass ihre Glaubensüberzeugung sich mit einer eGK mit Lichtbild nicht vereinbaren lässt, nicht. Ergänzend wies das BSG jedoch darauf hin, dass die Krankenkassen in diesen Fällen prüfen und entscheiden dürften, ob hinreichend substantiiert dargelegt ist, dass sich das Verhalten tatsächlich nach geistigem Gehalt und äußerer Erscheinung in plausibler Weise dem Schutzbereich des Art. 4 GG zuordnen lässt, also der Versicherte tatsächlich eine als religiös anzusehende Motivation hat.

### 3. *Bearbeitungsrecht der Krankenkasse*

Ist das eingereichte Bild „ungeeignet", muss geklärt werden, ob und inwieweit die Krankenkasse das zur Verfügung gestellte Lichtbild bearbeiten darf, um dessen Eignung herbeizuführen (das technische Können zunächst unterstellt). Diese Rechtsfrage berührt Belange, für deren Klärung Versicherten ein grundsätzliches Rechtsschutzbedürfnis nicht von vornherein abgesprochen werden kann[72]. Nach Art. 4 Nr. 2 DSGVO stellt u. a. die Anpassung oder Veränderung personenbezogener Daten eine datenschutzrelevante Verarbeitung dar.

Aus Sicht der Gerichte ist bei der Beurteilung des Lichtbildes die Eignung der eGK als Versicherungsnachweis gegen die geschützten Rechtsgüter der Versicherten abzuwägen, insbesondere gegen das allgemeine Persönlichkeitsrecht und das Recht auf informationelle Selbstbestimmung. Letztlich darf die Krankenkasse Bilder so bearbeiten, dass auf der eGK – entsprechend den o. g. Spezifikationen der gematik – nur noch das Gesicht des Versicherten zu sehen ist.

So bestätigte das SG Hamburg 2018 die Rechtmäßigkeit des Vorgehens einer Krankenkasse, die ein von Seiten des klagenden Versicherten zur Verfügung gestelltes Lichtbild, auf dem dieser unterhalb seines Kinns ein Schild mit seiner Versichertennummer hielt, so bearbeitete, dass zuletzt nur noch das Gesicht des Versicherten zu sehen war[73]. Mit der Bearbeitung habe die Krankenkasse einen legitimen gesetzlichen Zweck (s. o., Identifizierung zur Verhinderung missbräuchlicher Verwendung der eGK durch nichtversicherte Personen; Interesse an Vereinheitlichung) verfolgt; das persönliche Interesse eines Versicherten auf der eGK individuelle Botschaften unterzubringen, müsse insoweit zurückstehen[74]. Der Zuschnitt des Lichtbildes auf das Gesicht des Klägers sei auch geeignet, um die Eignung des Fotos für den Nachweiszweck sicherzustellen. Erforderlich war die Bearbeitung namentlich deshalb, weil der Kläger hinreichend deutlich machte, zur Einreichung eines anderen oder selbst bearbeiteten Lichtbildes nicht bereit zu sein[75]. Da der Grad der vorgenommenen Manipulation nur gering sei, war die Bearbeitung auch verhältnismäßig. Das Gesicht des Klägers selbst wurde nicht bearbeitet; der von der Beklagten abgeschnittene Teil des Bildes betrifft lediglich einen Gegenstand, nicht jedoch dessen äußeres Erscheinungsbild. Die Beeinträchtigung des Persönlichkeitsrechts des Klägers sei daher allenfalls minimal und ihm zumutbar. Der vom Kläger geltend gemachte Rechtsanspruch auf die Ausstellung einer eGK mit einer unbearbeiteten Version des eingereichten Bildes bestehe gerade nicht.

### 4. *Ablehnungs-/Zurückweisungsrecht der Krankenkasse*

Allem voran dann, wenn die Pflicht zu Einreichung faktisch dadurch zu umgehen versucht wird, dass ein ungeeignetes – nicht nachträglich entsprechend anzupassendes – Bild eingereicht wird, stellt sich die Frage, ob die Krankenkasse berechtigt ist, das eingereichte Lichtbild zurückzuweisen respektive die Ausstellung zu verweigern.

2020 entschied das SG Hamburg, dass Krankenkassen zur Verweigerung der Ausstellung einer eGK berechtigt sind, wenn Versicherte ungeeignete Lichtbilder, – hier solche, auf denen der Antragsteller jeweils ein Käppi oder eine Weihnachtsmütze auf dem Kopf trägt, – einreichen[76]. Es bestehe ein legitimes Interesse der Krankenkassen an einer Vereinheitlichung des Erscheinungsbildes der eGK. Das persönliche Interesse der Versicherten, auf der eGK die eigene Persönlichkeit und Meinung durch ein besonderes Erscheinungsbild zum Ausdruck zu bringen, müsse zurückstehen. Insbesondere Lichtbilder, die durch ihre Gestaltung geeignet sind, im Rechtsverkehr Zweifel an der Echtheit der eGK hervorzurufen, dürften von den Kassen zurückgewiesen werden.

Sichert die Krankenkasse die Ausstellung der eGK zu, erwächst hieraus auch kein Anspruch auf die Ausstellung einer eGK mit den anschließend eingereichten Bildern aus Zusicherung gem. § 34 Abs. 1 SGB X[77]. Ungeachtet des Schriftformerfordernisses komme eine Zusicherung nur bei Verwaltungsakten in Betracht. Die Ausstellung einer eGK sei aber nicht als Verwaltungsakt zu qualifizieren, sondern als Form tatsächlichen Verwaltungshandelns. Ob ein solcher Anspruch auf Ausstellung aus Erwägungen der Selbstverpflichtung von Behörden zu einem Realhandeln (mithin einer Zusage i. w. S.) erwachsen kann, stand demgegenüber bisher nicht zur (gerichtlichen) Entscheidung.

### 5. *Recht auf Ersatzbescheinigung*

Dem Kläger im eingangs (s. Teil I) genannten Verfahren vor dem LSG Bayern wurden vorab seiner Klageerhebung von seiner Krankenkasse insgesamt 13 eGK-Ersatzbescheinigungen – ohne Lichtbild – ausgestellt. Dies veranlasste ihn, die Ausstellung weiterer Ersatzbescheinigungen zu verlangen – gestützt auf die Annahme eines aus der vielfachen Ausstellung resultierenden Gewohnheitsrechts[78].

Dem folgte das LSG Bayern mit Verweis auf § 15 Abs. 6 S. 5 SGB V nicht. Danach kommt die wiederholte Ausstellung einer eGK-Ersatzbescheinigung (vgl. Satz 4 zur erstmaligen Ausstellung einer eGK-Ersatzbescheinigung bei Vorliegen vom Versicherten verschuldeter Gründe) nur in Betracht, wenn der Versicherte bei der Ausstellung der eGK mitwirkt; hierauf ist der Versicherte bei der erstma-

69) SG Stralsund, Beschl. v. 19. 10. 2015 – S 3 KR 235/15 ER –, juris, Rdnr. 17.
70) BSG, Urt. v. 19. 3. 2020 – B 1 KR 89/18 B –, juris, Rdnrn. 9 ff.; LSG Bad.-Württ., Urt. v. 24. 1. 2017 – L 11 KR 3562/16 –, juris, Rdnrn. 33 f.
71) BSG, Urt. v. 19. 3. 2020 – B 1 KR 89/18 B –, juris, Rdnr. 9. S. § 7 Abs. 3 S. 4 PAuswV. Die Personalausweisbehörde kann vom Verbot der Kopfbedeckung aus religiösen Gründen Ausnahmen zulassen.
72) S. nur SG Hamburg, Beschl. v. 14. 7. 2020 – S 30 KR 1024/20 ER –, juris, Rdnr. 11.
73) SG Hamburg, Urt. v. 17. 9. 2018 – S 21 KR 396/16 –, juris.
74) SG Hamburg, Urt. v. 17. 9. 2018 – S 21 KR 396/16 –, juris, Rdnrn. 25 ff.
75) SG Hamburg, Urt. v. 17. 9. 2018 – S 21 KR 396/16 –, juris, Rdnrn. 30 ff.
76) SG Hamburg, Beschl. v. 14. 7. 2020 – S 30 KR 1024/20 ER –, juris, Rdnrn. 2, 17 ff.
77) SG Hamburg, Beschl. v. 14. 7. 2020 – S 30 KR 1024/20 ER –, juris, Rdnr. 1 m. w. N.
78) Bay. LSG, Urt. v. 28. 9. 2021 – L 4 KR 651/19 –, juris, Rdnr. 7.

ligen Ausstellung einer Ersatzbescheinigung hinzuweisen. Verletzt der Versicherte durch die Nichtbeibringung eines aktuellen Lichtbildes seine Mitwirkungsobliegenheit, scheidet ein Anspruch auf eine zweite eGK-Ersatzbescheinigung somit dem eindeutigen Wortlaut nach aus. Begründete Zweifel an der Verfassungsmäßigkeit der Norm bestehen nicht.

Trotz des eindeutigen Wortlauts des § 15 Abs. 6 S. 5 SGB V verpflichtete das LSG Bayern die beklagte Krankenkasse im Verfahren des einstweiligen Rechtsschutzes indes, dem Antragsteller (Versicherten) eine befristete Ersatzbescheinigung auszustellen[79]. Bei summarischer Prüfung bestünden zwar nur äußerst geringe Erfolgsaussichten für das Hauptsacheverfahren. Mit der Verweigerung von grundsätzlichem Krankenversicherungsschutz gegenüber dem Versicherten durch die Nichtausstellung einer vorläufigen eGK-Ersatzbescheinigung wegen Nichtvorlage seines Lichtbildes verstoße die Antragsgegnerin jedoch gegen das rechtsstaatliche Gebot der Verhältnismäßigkeit (Art. 20 Abs. 3 GG). Der Antragsteller wäre hierdurch in seinem Grundrecht auf körperliche Unversehrtheit nach Art. 2 Abs. 2 S. 1 GG verletzt, da er keine ärztlichen Leistungen in Anspruch nehmen kann. Dies gelte zumindest für einstweilige Rechtsschutzverfahren, bei denen – wie hier – bei Gefahr des Entstehens schwerer und unzumutbarer, anders nicht abwendbarer Beeinträchtigungen eine umfassende Güter- und Folgeabwägung vorzunehmen sei.

### *6. Speicherung und Löschanspruch gem. § 291a Abs. 6 SGB V*

Dezidiert das Lichtbild auf der eGK betreffende Vorgaben zur Speicherung enthält seit 2020 § 291a Abs. 6 SGB V. Vor dessen Neufassung beschäftigte die Speicherung des Lichtbildes wiederholt die Gerichte, denn das Fehlen einer spezifischen Regelung zur Speicherung führte zu erheblichen Rechtsschwierigkeiten. Der nachstehende Vergleich deren Rechtsprechung zur alten Rechtslage mit dem Wortlaut des neuen § 291a Abs. 6 SGB V zeigt, dass der Gesetzgeber nicht vollständig mit der Ansicht (aller) Gerichte übereinstimmt.

Mit § 291a Abs. 6 SGB V existiert nun die erforderliche Rechtsgrundlage für die Krankenkassen, das Lichtbild der Versicherten für die Dauer der Mitgliedschaft, längstens für zehn Jahre für Ersatz- und Folgeausstellungen der eGK, zu speichern. Dies reduziert den Verwaltungsaufwand der Krankenkassen für Folgeausstattungen mit eGKn; zugleich wird dem Umstand Rechnung getragen, dass die intendierte Verbesserung der eindeutigen Zuordnung zum Versicherten zum Zwecke des Missbrauchsschutzes, mit einem mehr als zehn Jahre alten Lichtbild nicht mehr zuverlässig erreicht werden kann[80]. Nach dem Ende des Versicherungsverhältnisses hat die bisherige Krankenkasse das Lichtbild unverzüglich zu löschen, sobald der Versicherungsstatus abschließend geklärt ist – spätestens aber nach drei Monaten. Eine Löschung hat nur dann nicht zu erfolgen, wenn in dieser Zeit eine neue Versicherung beginnt. Ein Löschanspruch gleichen Inhalts folgt ebenfalls aus Art. 17 Abs. 1a DSGVO.

Der neu gefasste Absatz 6 berücksichtigt – in Teilen – die Rechtsprechung des BSG aus 2018, wonach eine kassenseitige dauerhafte Speicherung des Lichtbildes, d. h. bis zum Ende des Versicherungsverhältnisses, wegen der damals insoweit fehlenden Ermächtigungsgrundlage im Hinblick auf Art. 17 Abs. 1 lit. a DSGVO (datenschutzrechtlich) unzulässig war[81]. Vielmehr hätten Versicherte nach der Übermittlung einen Löschanspruch gegen die jeweilige Krankenkasse. Der in seinem Wortlaut bis heute unveränderte § 284 Abs. 1 S. 1 Nr. 2 SGB V[82] erlaubte aus damaliger Sicht des BSG Bilder für die eGK nur solange zu speichern, bis letztere hergestellt und so in den Herrschaftsbereich des Versicherten übermittelt worden war, dass dieser sie als Berechtigungsnachweis i. S. v. § 15 Abs. 2 SGB V verwenden kann[83]. Eine Ermächtigungsgrundlage, Bilder darüber hinaus zu speichern, fehle, denn die Erlaubnis zur Datenspeicherung beziehe sich schon dem Wortlaut nach bloß auf die Ausstellung einer einzigen konkreten („der") eGK, nicht hingegen auf eine Vorratsspeicherung für alle während eines Versicherungsverhältnisses auszustellenden Karten[84]. Herstellung und Übermittlung seien ein zeitlich abgrenzbarer Vorgang, kein Dauerzustand. Mit Abschluss der Übermittlung bedürfe die Krankenkasse der weiteren Lichtbildspeicherung „zur Ausstellung der eGK" nicht mehr. Das SG Berlin stellte in diesem Zusammenhang klar, dass auch kein Grund zu der Annahme bestehe, dass durch die Löschung schutzwürdige Interessen des Betroffenen beeinträchtigt werden (dies wäre z. B. zu bejahen, wenn zu erwarten ist, dass in zukünftigen Leistungsfällen auf die zwar aktuell nicht mehr benötigten, später aber nur noch schwer zu rekonstruierenden Sozialdaten zurückgegriffen werden muss).[85] Ein endgültiger Datenverlust drohe dank der jederzeit möglichen Übersendung (aktueller) Fotos jedoch gerade nicht.

Anders hatte dies zuvor das SG Mainz gesehen und – übereinstimmend mit der jetzigen Gesetzeslage und den dieser zugrunde liegen Erwägungen – eine Befugnis zur Speicherung des Lichtbildes zum Zweck der Erst- und Folgeausstellung der eGK bejaht[86]. Für die Speicherung im Zeitraum zwischen zwei Ausstellungen fehle es zwar an einer ausdrücklichen Regelung. Die Erforderlichkeit der Speicherung könne sich aber unter Beachtung der gesetzlichen Vorschriften aus einer Interessenabwägung zwischen dem Grundrecht auf informationelle Selbstbestimmung der Versicherten und den Erfordernissen einer Massenverwaltung ergeben. Der Bürokratieaufwand für die Kassen zur Neuanforderung von Lichtbildern (nach Ablauf der fünfjährigen Gültigkeit der eGK und im Verlustfall) bei rund acht Millionen Mitgliedern sei beträchtlich und daher von besonderem Gewicht. Zu erwägen sei, ob die Kassen den bürokratischen Aufwand durch Einwilligung der Versicherten zur Speicherung des Bildes für die gesamte Mitgliedschaftsdauer vermeiden könnten. Nach damaliger Gesetzeslage hätte die Kasse davon ausgehen dürfen, dass Versicherte so lange ein schutzwürdiges Interesse daran haben, so unbürokratisch wie möglich eine Ersatzkarte zu erhalten, bis ausdrücklich Löschung beantragt wird.

Hingegen ist nicht davon auszugehen, dass der § 291a Abs. 6 SGB V in der geltenden Fassung dispositiv ist. Denn angenommen, von der Norm könnte (zugunsten der Versicherten) abgewichen werden, würde dies bedeuten, dass der Gesetzgeber allein die Rechtsauffassung des BSG – sofortiger Löschanspruch gegenüber der GKV nach Abschluss der Übermittlung der eGK an den Versicherten – hat obsolet werden lassen, nicht aber die des SG Mainz, wonach bei rechtskonformer Einwilligung des Versicherten – in dessen Interesse – eine längere Speicherdauer, ggf. bis zur Grenze des gesamten Mitgliedschaftsverhältnisses,

79) Bay. LSG, Beschl. v. 20. 12. 2019 – L 4 KR 646/19 ER.
80) BT-Dr. 19/18793, 96, 112.
81) BSG, Urt. v. 18. 12. 2018 – B1 KR 31/17 R –, juris, Rdnrn. 20 f. Dazu schon *Ziebarth*, WzS 2016, 51.
82) Die Krankenkassen dürfen Sozialdaten für Zwecke der Krankenversicherung nur erheben und speichern, soweit diese für die Ausstellung des Berechtigungsscheines und der elektronischen Gesundheitskarte erforderlich sind.
83) A. A. LSG Bad.-Württ., Urt. v. 23. 11. 2016 – L 5 KR 1768/16 –, juris, Rdnr. 28 (Vorinstanz).
84) BSG, Urt. v. 18. 12. 2018 – B1 KR 31/17 R –, juris, Rdnrn. 20 f. m. w. N.
85) SG Berlin, Urt. v. 27. 6. 2017 – S 208 KR 2111/16 –, juris, Rdnr. 53 m. w. N.
86) SG Mainz, Pressemeldung 8/2015 v. 2. 12. 2015. Das Verfahren wurde durch Einigung zur Löschung beendet.

zu erwägen wäre (vorbehaltlich eines jederzeitigen Widerrufsrechts, vgl. Art. 7 Abs. 3 S. 1 DSGVO). Da die gesetzlich normierte Maximaldauer von 10 Jahren jedoch dem Gedanken der Identifizierung Rechnung trägt (optische Veränderung des Versicherten z. B. infolge Alters), ist eine Vereinbarung längerer Speicherfristen im Ergebnis aus teleologischen Erwägungen abzulehnen. Die ausdrückliche Vereinbarung kürzerer Fristen wäre hingegen zumindest mit dem Wortlaut vereinbar („längstens").

## V. „Gesundheitskarten" außerhalb der gesetzlichen Krankenversicherung

Soweit ersichtlich stehen alle veröffentlichten Gerichtsentscheidungen im Zusammenhang mit der GKV.

Die überwiegende Zahl der Unternehmen der privaten Krankenversicherung (PKV) gibt nach Beginn des Versicherungsschutzes – inzwischen[87] – ebenfalls eine „Versichertenkarte" aus (sog. Card für Privatversicherte/Klinik-Card). Eine Pflicht zur Nutzung der „Card" besteht indes nicht. Die Karte stellt lediglich ein Service-Angebot dar, das der Vereinfachung von Verwaltungsvorgängen dienen, den Kundenservice verbessern und den Status als „Privatpatient" erkennbar machen soll. Ungeachtet der Freiwilligkeit sieht die Card ohnehin kein Bild vor. Ein solches verpflichtend einzuführen (etwa durch Aufnahme eines Lichtbilderfordernisses in den AGB), dürfte wegen der in der PKV herrschenden Vertragsautonomie schwierig sein.

Vor seiner Neufassung durch das PDSG mit Wirkung zum 20. 10. 2020 sah § 291a Abs. 1a S. 1 SGB a. F. die entsprechende Anwendung des § 291a SGB V a. F. auf eGK privater Krankenversicherungsunternehmen vor. Diente die Kartenausgabe der Verarbeitung von Daten nach § 291a Abs. 3 S. 1 SGB V a. F., so unterlag sie v. a. im Hinblick auf ihre Nutzung sowie auf Datenschutz und Datensicherheit den für die eGK der GKV geltenden Beschränkungen[88].

Nachdem die eGK im GKV-Bereich Voraussetzung für die Nutzung der TI und von Fachdiensten wie der ePA und dem E-Rezept ist, werden PKV-Unternehmen, die seit Anfang 2022 die TI für ihre Versicherten nutzen möchten, jedenfalls dann eine eGK ausgeben müssen, soweit – mangels Vorhandensein einer digitalen Identität – TI-Anwendungen nicht mittels dieser als alternativem Zugriffsmittel in Anspruch genommen werden können (speziell für die ePA im Zusammenhang mit der PKV vgl. § 341 Abs. 5 SGB V). Nach Angaben des Verbandes der Privaten Krankenversicherung e. V. wird derzeit daran gearbeitet, eine (weitere) Lösung zu finden, die die eGK ersetzt[89].

## VI. Exkurs: Rechtsvergleich am Beispiel Österreich

In Österreich verfügt erstmals die neue Generation der „e-card", die seit dem 1. 1. 2020 ausgegeben wird, über ein Lichtbild des Versicherten (§ 31a Abs. 8 Allgemeines Sozialversicherungsgesetz, ASVG)[90]. Bei der e-card handelt es sich um die personenbezogene Chipkarte des elektronischen Verwaltungssystems der österreichischen Sozialversicherung (Kranken-, Unfall-, Pensions- sowie Arbeitslosenversicherung). Ziel war auch hier, ein Plus an Sicherheit zu gewähren und die Identitätsüberprüfung zu erleichtern. Die neue e-card wird den Versicherten automatisch nach Hause zugestellt, sofern ein Lichtbild aus österreichischem Reisepass, Personalausweis, Scheckkartenführerschein oder aus dem Fremdregister vorhanden ist, da die Sozialversicherung die Lichtbilder aus bestehenden Registern zur Verfügung gestellt bekommt. Kinder unter 14 Jahren, Personen ab 70 Jahren sowie Personen ab Pflegestufe 4 sind von der Lichtbildplicht ausgenommen (§ 31a Abs. 10 ASVG). Versicherte ohne hinterlegtes Lichtbild müssen der zuständigen Sozialversicherungsstelle seit dem 1. 1. 2020 ein Passfoto zur Verfügung stellen (§ 31a Abs. 9 ASVG). Für Nichtstaatsbürger ist die Landespolizeidirektionen zuständig. Bis 31. 12. 2023 sind alle e-cards, auf denen noch kein Lichtbild angebracht ist, auszutauschen. Die e-card ist eine Schlüsselkarte, die den Zugang zum e-card-System sowie zur elektronischen Gesundheitsakte ELGA eröffnet.

Soweit ersichtlich war der Österreichische Oberste Gerichtshof (OGH) mit dem in § 31a Abs. 8 ASVG normierten Lichtbilderfordernis bislang nicht befasst.

## VII. Rechtsschutz

Ob die datenschutzrechtlichen Vorgaben der DSGVO und des SGB V, namentlich die datenschutzrechtlichen Vorgaben im Zusammenhang mit der eGK und der TI, durch die dafür nach Art. 4 Nr. 7 DSGVO i. V. mit § 307 SGB V Verantwortlichen im Einzelnen eingehalten werden, ist durch die zuständigen Aufsichtsbehörden im Rahmen ihrer Aufgaben und Befugnisse nach Art. 57 f. DSGVO zu überwachen[91]. Versicherte können diese Einhaltung im Rahmen der speziellen datenschutzrechtlichen Rechtsbehelfe gem. Art. 77 ff. DSGVO i. V. mit §§ 81 ff. SGB X gerichtlich überprüfen lassen, ohne dass hierdurch die gesetzliche Obliegenheit zur Nutzung der eGK und deren Verfassungsmäßigkeit tangiert werden[92]. Generell ist das BSG bei der Annahme eines Rechtsschutzbedürfnisses trotz gefestigter (entgegenstehender) Rechtsprechung sehr großzügig[93].

Nach Art. 77 Abs. 1 DSGVO hat jede betroffene Person das Recht, sich bei der zuständigen Aufsichtsbehörde zu beschweren, wenn sie der Ansicht ist, dass die Verarbeitung der sie betreffenden personenbezogenen Daten gegen die DSGVO verstößt. Dementsprechend regelt § 81 Abs. 1 SGB X, dass sich eine betroffene Person, die der Ansicht ist, bei der Verarbeitung ihrer Sozialdaten in ihren Rechten verletzt worden zu sein, entweder an den Bundesbeauftragten oder die Bundesbeauftragte (Nr. 1) oder an die nach Landesrecht für die Kontrolle des Datenschutzes zuständige Stelle (Nr. 2) wenden kann. Gegen die daraufhin ergehende Entscheidung, wie auch gegen die Untätigkeit der genannten Stellen kann die betroffene Person nach Art. 78 DSGVO i. V. mit § 81a SGB X bzw. § 20 BDSG gerichtlichen Rechtsschutz in Anspruch nehmen. Darüber hinaus besteht für die betroffene Person nach Art. 79 DSGVO i. V. mit § 81b SGB X auch das Recht, unmittelbar gegen den oder die datenschutzrechtlich Verantwortlichen und/oder Auftragsverarbeiter gerichtlich vorzugehen, wenn sie der Ansicht ist, dass die ihr aufgrund dieser Verordnung zustehenden Rechte infolge einer nicht im Einklang mit dieser Verordnung

87) Anders als ursprünglich geplant, erfolgte die bundesweite Einführung der eGK zunächst ohne die Privatversicherten. Hintergrund dessen war, dass für Leistungserbringer keine verpflichtende Anwendung der Karte von Privatpatienten bestand.

88) *Michels*, in: *Becker/Kingreen*, SGB V, 7. Aufl. 2020, Rdnr. 3. Weil private Krankenversicherungsunternehmen nicht am Aufbau und der Finanzierung der TI beteiligt waren und nicht dem Anwendungsbereich der VO 883/2004 unterliegen, fanden § 291a Abs. 5 und Abs. 7 SGB V a. F. keine entsprechende Anwendung.

89) https://www.pkv.de/verband/presse/meldungen-2021/pkv-und-e-health-wir-wollen-neue-digitale-anwendungen-mobil-und-smart-gestalten/.

90) Art. 1 des Sozialversicherungs-Zuordnungsgesetzes (SV-ZG), BGBl. I Nr. 125/2017. Dort ist in Abs. 8 allerdings noch vom 1. 1. 2019 die Rede. Vgl. ebenfalls BGBl. I Nr. 23/2019 sowie die e-card FotoV, BGBl. II Nr. 231/2019. Zur e-Card *Riedl/Schönfeld*, SozSich Öst 2019, 391.

91) BSG, Urt. v. 20. 1. 2021 – B 1 KR 7/20 R –, juris, Rdnr. 100. Im Einzelnen: *Bieresborn/Giesberts-Kaminski*, SGb 2018, 609; *Leopold*, ZESAR 2018, 326.

92) BSG, Urt. v. 20. 1. 2021 – B 1 KR 7/20 R –, juris, Ls. 3.

93) BSG, Urt. v. 18. 11. 2014 – B 1 KR 35/13 R –, juris, Rdnr. 12.

stehenden Verarbeitung ihrer personenbezogenen Daten verletzt wurden.

Die Einhaltung der Datensicherheit ist durch die Datenschutzkontrolle nach §§ 329, 333 SGB V als institutionelle Sicherung einerseits und die Straf- und Bußgeldvorschriften nach §§ 397 ff. SGB V andererseits, gewährleistet[94].

Der Gesetzgeber ist mit den Neureglungen folglich auch seiner Beobachtungs- und Nachbesserungspflicht nachgekommen, indem er u. a. auf die in der Praxis zutage getretenen datenschutzrechtlichen Defizite und Sicherheitsmängel mit entsprechenden Gegenmaßnahmen reagiert hat[95].

Mit Blick auf die zur Verfügung stehenden speziellen datenschutzrechtlichen Rechtsbehelfe, verneinten die Gerichte das Erfordernis einer Vorlage der streitbehafteten Normen an den EuGH oder das BVerfG[96].

## VIII. Ausblick: Digitale Identität für das Gesundheitswesen gem. § 291 Abs. 8 SGB V

Digitale Identitäten sind elementarer Bestandteil des gesellschaftlichen Gefüges und Indiz erfolgreich umgesetzter Digitalisierung. Das gilt auch für den Gesundheitssektor. Spätestens zum 1. 1. 2023 müssen die Krankenkassen – auf Verlangen des Versicherten – diesem ergänzend zur eGK barrierefrei eine sichere digitale Identität zur Verfügung stellen (§ 291 Abs. 8 S. 1 SGB V), die die Vorgaben nach § 291 Abs. 2 Nr. 1 und 2 SGB V erfüllt (d. h. technische Eignung, Authentifizierung, Verschlüsselung und elektronische Signatur barrierefrei zu ermöglichen sowie die Anwendungen der Telematikinfrastruktur nach § 334 Abs. 1 SGB V zu unterstützen) und die Bereitstellung von Daten nach § 291a Abs. 2 und 3 SGB V durch die Krankenkassen ermöglicht. Ab dem 1. 1. 2024 dient die digitale Identität in gleicher Weise wie die eGK zur Authentisierung des Versicherten im Gesundheitswesen und als Versicherungsnachweis nach § 291a Abs. 1 SGB V, ohne an eine Chipkarte gebunden zu sein (§ 15 Abs. 2 S. 2 SGB V; § 291a Abs. 1 S. 3 SGB V). Darüber hinaus ist sie v. a. in der digitalen Gesundheitsvorsorge relevant. Spätestens seit dem 1. 7. 2022 stellen die Kassen berechtigten Dritten Verfahren zur Erprobung der Integration der sicheren digitalen Identität zur Verfügung (§ 291 Abs. 8 S. 7 SGB V).

Hintergrund der Neuregelung ist das Digitale–Versorgung–und–Pflege–Modernisierungs–Gesetz (DVPMG), das seit dem 9. 6. 2021 gilt und u. a. das SGB V (dieses besonders umfangreich) ändert. Ziel ist es, die Gesundheitsversorgung zu digitalisieren, v. a. die Versorgung mit digitalen Gesundheits-, speziell digitalen Pflegeanwendungen auszubauen[97]. Mit dem DVPMG wurden sowohl die gesetzlichen Voraussetzungen geschaffen als auch Anreize gesetzt, die vom Gesetzgeber verfolgten Ziele in der Praxis zu realisieren.

Nach § 291 Abs. 8 S. 5 und 6 SGB V kann eine digitale Identität über überschiedliche Ausprägungen mit verschiedenen Sicherheits- und Vertrauensniveaus verfügen, allerdings muss letzteres mindestens dem Schutzbedarf der Anwendung entsprechen, bei der diese eingesetzt wird. § 291b Abs. 7 SGB V ermächtigt das BMG, die in § 291b Abs. 1 und 2 und § 291 Abs. 2 Nr. 3 und Abs. 8 SGB V genannten Fristen durch Rechtsverordnung ohne Zustimmung des Bundesrates zu verlängern. Vorab hat die gematik jedoch Festlegungen im Hinblick auf Sicherheit und Interoperabilität zu treffen (§ 291 Abs. 8 S. 3 SGB V).

## IX. Fazit

Das zu Beginn (s. Teil I) genannte Urteil des LSG Bayern bestätigt die Entscheidungen der Gerichte der Vergangenheit, speziell des BSG: Das geltende Recht zur Ausgestaltung und Verwendung der eGK ist verfassungskonform. Das Spannungsverhältnis zwischen begründeten Interessen an der Eignung der eGK als Versicherungsnachweis und den verfassungsrechtlich geschützten Rechtsgütern der Versicherten ist nach der Rechtsprechung im Ausgangspunkt zugunsten Ersterem aufzulösen. Diese Auffassung vertritt offenkundig auch der Gesetzgeber, wie die oben dargestellten Neuregelungen belegen. Gleichwohl müssen die Krankenkassen nach der sozialgerichtlichen Rechtsprechung sowohl bei der Bearbeitung der eingereichten Fotos als auch konkret der Anwendung von § 291a Abs. 5 SGB V selbstredend die Persönlichkeitsrechte der Versicherten berücksichtigen[98].

Weitere Ermittlungen von Amts wegen, v. a. durch Einholung der Stellungnahme eines IT-Experten, waren daher auch im Verfahren vor dem LSG Bayern nicht angezeigt.[99] Eine Vorlage des Rechtsstreits an das BVerfG nach Art. 100 Abs. 1 GG schied aus den gleichen Gründen aus.

Die Frage nach der Rechtskonformität, spezifisch der (die DSGVO in den Blick nehmenden) Datenschutzkonformität, des Lichtbildes auf der eGK wird die Gerichte, trotz deren eindeutiger wie beständig einheitlicher Position, sehr wahrscheinlich auch in Zukunft noch beschäftigen – nicht zuletzt deshalb, weil das BSG (s. o.) ausdrücklich ein Rechtsschutzbedürfnis all jener Versicherten bejaht, die sich durch das Erfordernis der Verwendung einer eGK mit ihren Angaben zur Person, den deutlich erweiterten technischen Möglichkeiten und dem Lichtbilderfordernis in ihren Rechten verletzt sehen. Überdies ist das sozialgerichtliche Verfahren für Versicherte im Regelfall kostenlos bzw. stark kostenreduziert, §§ 192 f. SGG.

In Anbetracht der (derzeit) gleichwohl eher geringen Erfolgsaussichten einer Klage gegen das Lichtbilderfordernis sei zumindest der Vollständigkeit halber erwähnt, dass es einem jeden privaten Verbraucher, der die Lichtbildpflicht der GKV umgehen will, freisteht, in die PKV zu wechseln. Seit dem 1. 1. 2009 müssen private Krankenkassen einen sog. „Basistarif" anbieten, für den wie bei gesetzlichen Krankenkassen ein Kontrahierungszwang gilt (vgl. v. a. § 193 Abs. 5 VVG und § 12 Abs. 1b VAG). Indem das BSG die Frage, ob Versicherte die Verwendung der eGK wegen ihrer Online-Funktion und der ihr zugrundeliegenden TI wirksam ablehnen können, eindeutig verneinte[100], besteht für Versicherte hingegen gerade keine Möglichkeit, das Fotoerfordernis mit dieser Argumentation „zu umgehen".

Zwar steht es Versicherten spätestens ab 2024 frei, sich für die sichere digitale Identität zu entscheiden. Der Streit um das Lichtbild auf der eGK wird dadurch aber wohl kaum in den Hintergrund rücken: einmal, weil die digitale Identität Versicherten nur ergänzend zur eGK zur Verfügung stehen wird, vor allem aber, weil die digitale Identität zuvorderst eine Alternative für die TI-Anwendungen darstellt – und

94) Vgl. *Schiffdecker*, in: KassKomm Sozialversicherungsrecht, 118. EL März 2022, § 291 SGB V, Rdnr. 24.

95) BSG, RDG 2021, 185.

96) Statt vieler: BSG, Urt. v. 20. 1. 2021 – B 1 KR 7/20 R –, juris, Rdnr. 14.

97) BT-Dr. 19/27652, 2. Künftig hat die gematik das Recht, Komponenten und Dienste der Telematikinfrastruktur selbst zu betreiben sowie darf die Sicherheits- und Interoperabilitätsanforderungen an die digitalen Identitäten festlegen (§ 291 Abs. 8 S. 3 SGB V), wobei die Festlegung der Anforderungen an die Sicherheit und den Datenschutz im Einvernehmen mit dem BSI und der oder dem BfDI auf Basis der jeweils gültigen TR des BSI und unter Berücksichtigung der notwendigen Vertrauensniveaus der unterstützten Anwendungen erfolgt (§ 291 Abs. 8 S. 4 SGB V).

98) BSG, Beschl. v. 19. 3. 2020 – B 1 KR 89/18 B –, juris, Rdnr. 12 m. w. N.

99) Bay. LSG, Urt. v. 28. 9. 2021 – L 4 KR 651/19 –, juris, Rdnrn. 47 f. mit Verweis auf BSG, Urt. v. 20. 1. 2021 – B 1 KR 7/20 R –, juris, Rdnr. 116.

100) BSG, Urt. v. 20. 1. 2021 – B 1 KR 7/20 R –, juris.

daher die Pflicht zur Vorlage der eGK – mit Lichtbild – zu Identifizierungszwecken unberührt lassen wird (vgl. § 291 Abs. 1 SGB V).

Im Ergebnis ist die sukzessive Digitalisierung des Gesundheitssektors zweifellos als Mehrwert, allem voran aber als wichtiger Schritt zu begreifen, bei dem datenschutz- und verfassungsrechtliche Zweifel oftmals unvermeidbar zugunsten einer flächendeckenden, effizienten sowie höchste technische wie medizinische Standards gewährende Gesundheitsversorgung zurücktreten müssen.

**Open Access.** Dieser Artikel wird unter der Creative Commons Namensnennung 4.0 International Lizenz veröffentlicht, welche die Nutzung, Vervielfältigung, Bearbeitung, Verbreitung und Wiedergabe in jeglichem Medium und Format erlaubt, sofern Sie den/die ursprünglichen Autor(en) und die Quelle ordnungsgemäß nennen, einen Link zur Creative Commons Lizenz beifügen und angeben, ob Änderungen vorgenommen wurden.
Die in diesem Artikel enthaltenen Bilder und sonstiges Drittmaterial unterliegen ebenfalls der genannten Creative Commons Lizenz, sofern sich aus der Abbildungslegende nichts anderes ergibt. Sofern das betreffende Material nicht unter der genannten Creative Commons Lizenz steht und die betreffende Handlung nicht nach gesetzlichen Vorschriften erlaubt ist, ist für die oben aufgeführten Weiterverwendungen des Materials die Einwilligung des jeweiligen Rechteinhabers einzuholen.
Weitere Details zur Lizenz entnehmen Sie bitte der Lizenzinformation auf http://creativecommons.org/licenses/by/4.0/deed.de.

Funding

Open access funding enabled and organized by Projekt DEAL.

